# Lung recruitment strategies

**DOI:** 10.1186/1824-7288-41-S1-A13

**Published:** 2015-09-24

**Authors:** Paolo Gancia, Giulia Pomero

**Affiliations:** 1 Terapia Intensiva Neonatale-Neonatologia, ASO S. Croce e Carle, Cuneo, Italy

## 

Mechanical ventilation in newborns, children and adults leads to lung injury and has been shown to induce the formation of proteinaceous lung oedema causing epithelial disruption and resulting in changes in lung perfusion. Lung injury leads to reduced compliance, worsening of shunt fraction and an inflammatory response that results from high end-inspiratory transpulmonary pressures (Ptp) and inadequate End Expiratory Lung Volume (EELV). Lung injury results if a lung is ventilated from collapsed state with each ventilator cycle. Thus the first concept of treatment in acute hypoxic respiratory failure is: “Open collapsed lung units and keep them open without overdistending them”. The open lung is one in which there is little or no atelectasis and an optimal gas exchange. Lung recruitment strategies transiently increase Ptp to reopen the alveolar units not aerated or poorly aerated but recruitable.

Lung recruitment maneuvers may restore EELV resulting in more stable alveoli, and reduce shear stress associated with the alveolar cyclic opening and closing. Lung recruitment can be performed in several ways: in delivery room with a T-piece device, in the NICU setting by using a conventional or high frequency oscillation (HFO) ventilator, and is commonly achieved by increase of end-expiratory lung volume with positive end expiratory pressure (PEEP) or end inspiratory lung volume by inspiratory holds or sustained inflation. Recruitment during both, HFO and CMV (Conventional Mechanical Ventilation), follows similar concepts when using small tidal volume ventilation but is easier to achieve at bedside during HFO.

The Sustained Inflation maneuver (SI) applies a high pressure to the lung for a short period (30 sec) before returning to previous mean airway pressures. This volume recruitment strategy has been shown to be as protective as high frequency oscillatory ventilation (HFO) at similar lung volumes. Infants treated with a SI in delivery room had improved short-term respiratory outcomes (reduced need of tracheal intubation and mechanical ventilation), but major outcomes (BPD Bronchopulmonary dysplasia*-* and/or death) were not improved.

Recent reports describe improvements in arterial oxygenation with the use of recruitment maneuvers during CMV and/or HFO. However, the impact of these strategies on patient important outcomes such as survival is still unclear.

Further clinical studies are needed to evaluate the efficacy of SI, including selection of patients, setting of SI maneuver in terms of duration and peak pressure, timing of surfactant administration. Further studies are also needed to elucidate the impact of recruitment maneuvers during mechanical ventilation, including survival and BPD.
